# Supplementary material for: Identification of Freezing-Responsive microRNAs and Their Targets in Chinese Jujube by Small RNA and Degradome Sequencing
Source: Int J Mol Sci. 2026 Sep 4;27(17):7912. doi: 10.3390/ijms27177912 (PMC13566890; doi:10.3390/ijms27177912)
Supplement: Supplementary file 1 [file ijms-27-07912-s001.zip › Supplementary Material S2/novel_miR_8.pdf]

Provisional ID : novel_miR_8
Score total : 186.5
Score for star read(s) : 3.9
Score for read counts : 180.6
Score for mfe : 0.4
Score for randfold : 1.6
Score for cons. seed :
Total read count : 366
Mature read count : 249
Loop read count : 0
Star read count : 117

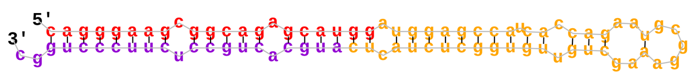

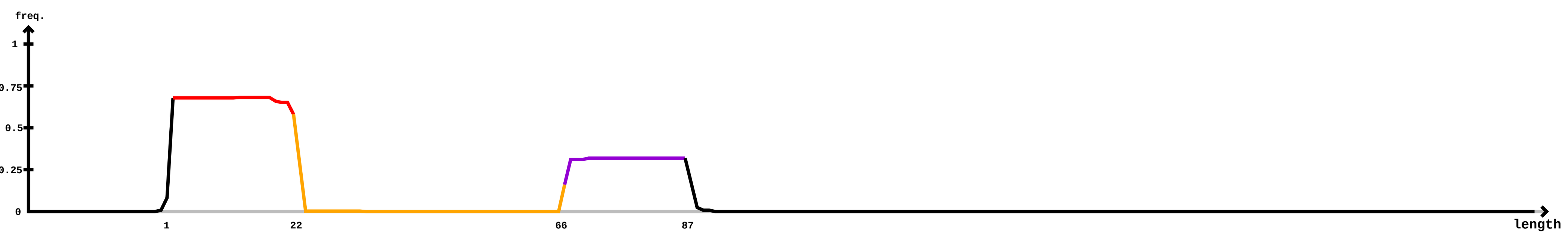

Mature Star

```
5'- auggguuuggaaaauaaaaagacagggaagcggcagagcauggauggagccaucaccagaaugcggaaagcuguuguggcucuacucaugcacugccucuucccuggcuugccauaucauaucauuccucucuuucuuuuucccuccuaauucauuuucuuauuuaucuauuuauuuuuaauuaacucacucuugcuuugguacauaauauuuuuuuuuccccuuuucuuuauuacuuuuggaggcac   -3'   obs
    auggguuuggaaaauaaaaagacagggaagcggcagagcauggauggagccaucaccagaaugcggaaagcuguuguggcucuacucaugcacugccucuucccuggcuugccauaucauaucauuccucucuuucuuuuucccuccuaauucauuuucuuauuuaucuauuuauuuuuaauuaacucacucuugcuuugguacauaauauuuuuuuuuccccuuuucuuuauuacuuuuggaggcac         exp
    (((((((.(((....((((((((((((((.(((((.((((((.((((((((.((.(((..(.....)..))).)))))))))))).)))))).))))).))))))))........................)))))))...))).))))))))[illegible]((((((.(....(((((......................))))).....).))))))).   reads   mm   sample
```

read	reads	mm	sample
....................Cacagggaagcggcagagc...	3	1	LCK-3
.....................acagggaagcggcagagc...	3	0	LT-1
.....................acagggaagcggcagagc...	1	0	JCK-1
.....................acagggaagcggcagagc...	1	0	LT-3
.....................acagggaagcggcagagca...	1	0	LCK-3
.....................acagggaagcggcagagcaug...	1	0	JCK-2
.....................acagggaagcggcagagcaug...	1	0	LT-3
.....................acagggaagcggcagagcaug...	1	0	LT-1
.....................acagggaagcggcagagcaug...	13	0	LCK-3
.....................acagggaagcggcagagcaug...	3	0	LT-2
.....................acagggaagcggcagagcaug...	1	0	JCK-3
.....................acagggaagcggcagagcaugU...	1	1	JCK-1
......................cagggaagcggcagagca...	2	0	LT-1
......................cagggaagcggcagagcaug...	4	0	JCK-2
......................cagggaagcggcagagcaug...	2	0	JCK-3
......................cagggaagcggcagagcaugg...	25	0	LCK-3
......................cagggaCgcggcagagcaugg...	2	1	JCK-2
......................cagggaagcggcagagcaugg...	12	0	LT-2
......................cagggaagcggcagagcaugg...	4	0	LT-1
......................cagggaagcggcagagcaugg...	12	0	LT-3
......................cCgggaagcggcagagcaugg...	1	1	JCK-3
......................cagggaagcggcagagcaugg...	62	0	JCK-2
......................cagggaagcggcagagcaugU...	4	1	LT-2
......................cagggaagcggcagagcaugg...	48	0	JCK-3
......................cagggaagcggcagagcaugg...	39	0	JCK-1
......................cagggaagcggUagagcaugg...	1	1	JCK-2
......................cagggaagcggcagagcaugga...	1	0	LT-1
................................cagagcauggauggagccauc...	1	0	JCK-1
...augcacugccucuucccuggc...	3	0	JCK-2
...Cugcacugccucuucccuggc...	17	1	JT-1
...augcacugccucuucccuggc...	8	0	LT-3
...augcacugccucuucccuggc...	3	0	LT-1
...augcacugccucuucccuggc...	2	0	LCK-3

```
             Mature                                                Star
augguuuggaaaauaaaaagacagggaagcggcagagcauggauggagccaucaccagaaugcggaaagcuguuguggcucuacucaugcacugccucuucccuggcuugccauaucauaucauuccucucuuucuuuuucccuccuaauucauuuucuuauuuaucuauuuauuuuuaauuaacucacucuugcuuugguacauaauauuuuuuuuuuccccuuuucuuuauuacuuuuggaggcac
.......................augcacugccucuucccuggc.......................   3    0    JCK-3
.......................augcacugccucuucccuggc.......................   3    0    JCK-1
.......................Cugcacugccucuucccuggc.......................   1    1    LT-3
.......................augcacugccucuucccuggc.......................   19   0    LCK-2
........................ugcacugccucuucccuggc.......................   1    0    LT-1
........................ugcacugccucuucccuggc.......................   1    0    LT-3
........................ugcacugccucuucccuggc.......................   5    0    JCK-2
........................ugcacugccucuucccuggU.......................   1    1    LCK-2
........................ugcacugccucuucccuggc.......................   1    0    LCK-2
........................ugcacugccucuucccuggc.......................   5    0    JCK-1
........................ugcacugccucuucccuggc.......................   22   0    JT-1
........................ugcacugccucuucccuggc.......................   8    0    JCK-3
........................ugcacugccucuucccuggcu......................   1    0    JCK-2
........................ugcacugccucuucccuggcu......................   1    0    LCK-2
........................ugcacugccucuucccuggcu......................   3    0    LT-2
........................ugcacugccucuucccuggcuu.....................   1    0    JCK-1
........................ugcacugccucuucccuggcuu.....................   1    0    JCK-3
........................ugcacugccucuucccuggcuC.....................   2    1    LCK-2
........................ugcacugccucuucccuggcuu.....................   1    0    JCK-2
........................ugcacugccucuucccuggcuu.....................   1    0    LCK-2
...........................acugccucuucccuggcuugc...................   2    0    JCK-2
...........................acugccucuucccuggcuugc...................   1    0    JCK-1
```
